# Rhamnogalacturonan-I as a nematode chemoattractant from *Lotus corniculatus* L. super-growing root culture

**DOI:** 10.3389/fpls.2022.1008725

**Published:** 2023-01-26

**Authors:** Morihiro Oota, Syuuto Toyoda, Toshihisa Kotake, Naoki Wada, Masatsugu Hashiguchi, Ryo Akashi, Hayato Ishikawa, Bruno Favery, Allen Yi-Lun Tsai, Shinichiro Sawa

**Affiliations:** ^1^ Faculty of Advanced Science and Technology, Kumamoto University, Kumamoto, Japan; ^2^ Division of Life Science, Graduate School of Science and Engineering, Saitama University, Saitama, Japan; ^3^ Faculty of Regional Innovation, University of Miyazaki, Miyazaki, Japan; ^4^ Faculty of Agriculture, University of Miyazaki, Miyazaki, Japan; ^5^ Graduate School of Pharmaceutical Sciences, Chiba University, Chiba, Japan; ^6^ Institut national de recherche pour l'agriculture, l'alimentation et l’environnement (INRAE), Université Côte d’Azur, CNRS, UMR 1355-7254 Institut Sophia Agrobiotech, Sophia Antipolis, France; ^7^ International Research Organization for Advanced Science and Technology, Kumamoto University, Kumamoto, Japan; ^8^ International Research Center for Agricultural and Environmental Biology, Kumamoto University, Kumamoto, Japan

**Keywords:** root-knot nematodes (*Meloidogyne incognita*), super roots, chemotaxis, pectin, *Lotus corniculatus* L.

## Abstract

**Introduction:**

The soil houses a tremendous amount of micro-organisms, many of which are plant parasites and pathogens by feeding off plant roots for sustenance. Such root pathogens and parasites often rely on plant-secreted signaling molecules in the rhizosphere as host guidance cues. Here we describe the isolation and characterization of a chemoattractant of plant-parasitic root-knot nematodes (*Meloidogyne incognita*, RKN).

**Methods:**

The Super-growing Root (SR) culture, consisting of excised roots from the legume species *Lotus corniculatus* L., was found to strongly attract infective RKN juveniles and actively secrete chemoattractants into the liquid culture media. The chemo-attractant in the culture media supernatant was purified using hydrophobicity and anion exchange chromatography, and found to be enriched in carbohydrates.

**Results:**

Monosaccharide analyses suggest the chemo-attractant contains a wide array of sugars, but is enriched in arabinose, galactose and galacturonic acid. This purified chemoattractant was shown to contain pectin, specifically anti-rhamnogalacturonan-I and anti-arabinogalactan protein epitopes but not anti-homogalacturonan epitopes. More importantly, the arabinose and galactose sidechain groups were found to be essential for RKN-attracting activities. This chemo-attractant appears to be specific to *M. incognita*, as it wasn’t effective in attracting other *Meloidogyne* species nor *Caenorhabditis elegans*.

**Discussion:**

This is the first report to identify the nematode attractant purified from root exudate of *L corniculatus* L. Our findings re-enforce pectic carbohydrates as important chemicals mediating micro-organism chemotaxis in the soil, and also highlight the unexpected utilities of the SR culture system in root pathogen research.

## Introduction

Root-knot nematodes (RKN, *Meloidogyne incognita*) are plant pathogens that infect many plant species, including economically important crops, causing up to several billions of USD worth of agricultural damage annually (Singh et al., 2013). In the soil, RKN molt once inside the egg and hatch as second-stage juveniles (J2), which migrate freely to seek out appropriate host plant roots to infect. Once a J2 has found a suitable host, it enters the root near the root tip. It then migrates to the vasculature, where it remains sedentary for the rest of its life. The J2 then injects various effector proteins into the host cells, which hijack the host cells’ developmental program and convert them into multinucleated giant cells to serve as the J2’s sustenance (Mejias et al., 2019; Favery et al., 2020; Jagdale et al., 2021). These giant cells cause the infected regions of the root to swell and form the eponymous root knots or galls. The J2 then molt three more times to become adult females, which then emerge to the root surface to lay eggs and initiate the next generation. RKN infection and gall formation not only cause superficial deformities on the roots of host plants but also siphon nutrients from the host and disrupt metabolite transport, leading to poor growth, reduced yield, and even death of the host plant in severe cases (Singh et al., 2013).

During the RKN life cycle, J2 is the only stage where the RKN is free-living and not associated with host plants. Nevertheless, RKN J2s must find the roots of a suitable host plant to infect before their stored nutrients are depleted. Exudates from all root epidermal cells can be found in the soil, although root exudates are most prominently secreted by the root cap border cells and border-like cells, which are constantly shed as the root grows. These cells remain viable after becoming detached and continue to secrete a mixture of carbohydrates, proteins, and extra-cellular DNA to mediate interactions with soil microbes (Driouich et al., 2021). Studies have shown that RKN are attracted to various compounds in root exudates in order to find appropriate host plants (Curtis, 2008; Reynolds et al., 2011; Liu et al., 2019). Such behavior is known as chemotaxis, which refers to animal movements along a chemical concentration gradient and is critical not only for foraging but also for predator avoidance and courtship. Clearly, the perception of and response to chemoattractants is critical to the survival of RKN. However, currently the identities and importance of RKN attractants in root exudates remain to be deciphered. Mannitol, amino acids, arginine, lysine, phytohormones, salicylic acid, and indole-acetic acid (IAA) have all been shown to attract RKN J2s (Fleming et al., 2017). Since these metabolites can all be found in *Arabidopsis thaliana* root exudate, RKN may perceive these compounds as host-finding cues. Other RKN attractants identified from root exudates include volatiles (α-pinene, limonene, 2-methoxy-3-(1-methylpropyl)-pyrazine, methyl salicylate, and tridecane) from pepper (*Capsicum annum*), phytohormone (zeatin/cytokinin), flavonoids (quercetin and luteolin) and alkaloids (solasodine and tomatidine) from tomato (*Solanum lycopersicum*), and organic amines (cadaverine, putrescine, 1,3-diaminopropane) from soybean (*Glycine max*) (Kihika et al., 2017; Kirwa et al., 2018; Oota et al., 2020). However, it remains difficult to comprehensively explain RKN infection behavior and its wide host range with the current collection of known RKN attractants alone. Interestingly, RKN attractants were also identified in plant organs other than roots. RKN J2 were found to be attracted to Arabidopsis seeds in a seed coat mucilage extrusion-dependent manner, and L-Gal substituted rhamnogalacturonan-I in flaxseed mucilage (Tsai et al., 2019; Tsai et al., 2021). These lines of evidence suggest cell wall polysaccharides may also play a role in RKN chemotaxis.

As obligate parasites, RKN cannot be cultivated without their host. Plant tissue cultures represent an excellent alternative to growing whole plants, such as Arabidopsis and tomato models. These plants may require long growth times, for which it may be difficult to access infected tissue. Root tissue cultures thus emerge as promising candidates for further examination of RKN infection. With fast generation times, easy access to observations, and simple manipulation under aseptic conditions, tissue cultures may complement the shortcomings of whole-plant models. The super-growing root (SR) line of bird’s-foot trefoil (*Lotus corniculatus* L.) appears to be particularly successful at recreating root tissues under natural conditions. SR was initially isolated during routine root culture preparation and possessed particularly robust root growth activity and longevity, without exogenous phytohormone applications (Akashi et al., 1998; Akashi et al., 2003). As a result, SR was indeed useful in identifying genes that regulate root growth and rhizobia-induced nodulation (Tanaka et al., 2008; Jian et al., 2009; Himuro et al., 2011; Arifin et al., 2019). Nevertheless, the utility of SR in plant–pathogen interactions remains to be explored.

Here we present a novel RKN attractant secreted by SR culture and identify the attractant as a pectin-based derivative with Ara and Gal sidechains being essential for attraction. Our results highlight the importance of cell wall carbohydrates in RKN chemotaxis in roots. In addition, they highlight the potential of SR culture as a model system for RKN infection analysis.

## Materials and methods

### Nematodes and plant materials

*M. incognita* was isolated from Koshi City (Kumamoto, Japan) and cultivated as described by Nishiyama et al. (2015). *M. arenaria* (Guadeloupe strain) and *M. enterolobii* (Godet strain) come from the ISA collection of RKN strains (INRAE, Université Côte d’Azur, CNRS, Sophia Antipolis, France). *Caenorhabditis elegans* was kindly provided by Kunitoshi Yamanaka (Kumamoto University).

Super-growing root (*L. corniculatus* L.) culture was propagated as described by Akashi et al. (1998), with 28-day cultures used for attraction experiments. To test for RKN attraction, 5-day-old seedlings of Arabidopsis (*A. thaliana*, Col-0), tomato (*S. lycopersicum*, Pritz), and 6-day rice (*Oryza sativa*, Taichu-65) were used.

### Preparation of plant attractant

SR cultures were cultivated as described by Akashi et al. (1998). Forty *L. corniculatus* L. lateral roots from a previous culture were placed in 20 ml of liquid medium and incubated for 28 days. The liquid medium was collected and concentrated 10-fold using a freeze-dryer and used to check nematode attraction activity.

To purify the nematode attractants from the super-growing root culture medium, 900 ml of SR liquid medium was first mixed with equal volumes of ethyl acetate, and the aqueous fraction was then recovered. The process was repeated with an equal volume of ether. The aqueous fraction was then washed with ~500 ml of MeOH. The attractant was then further purified by hydrophobic chromatography using a SepPak C18 column (Waters, WAT043345), washed with 80 ml ddH_2_O, and eluted with 80 ml of 60% CH_3_CN. The 60% CH_3_CN eluate was then further purified with anion exchange (DEAE-cellulose, ChemCruz sc-506208) chromatography and eluted with 10 ml of ddH_2_O, 50 mM NaHCO_3,_ and 500 mM NaHCO_3_ to obtain the purified attractant.

### Nematode attraction assay

Attraction tests for *Meloidogyne* species were performed essentially as described in Tsai et al. (2019). Assays were performed on 32% Pluronic F-127 with 20,000 nematode J2 larvae in 60-mm petri dishes (Wang et al., 2009). The chemotaxis index was calculated using a method described in Tsai et al. (2019), with the formula:


$$Chemotaxis\;index=\frac{\left[sum(\#attracted)-sum(\#background)\right]}{\left[sum(\#total)\right]}$$

Attraction tests for *C. elegans* were performed essentially as described in Yoshimizu et al. (2018). Assays were performed on 1.5% agar supplemented with 5 mM potassium phosphate at pH 6.0, 1 mM CaCl_2_, and 1 mM MgCl_2_ in 9 cm petri dishes. Approximately 1 µl of the attractant and the cognate negative control were placed at opposing ends of the petri dish. Approximately 1 µl droplets of 1 M sodium azide were placed on the lid above the attractant and negative control to immobilize *C. elegans*. Used as a positive control was 1% isoamyl alcohol in ethanol. This was incubated at 20°C for 1 h. The chemotaxis index was calculated as described in Tsai et al. (2021).

### Microscopy

The attraction of the nematodes was imaged with an AxioZoom V16 dissecting microscope (Zeiss) mounted with a DP74 digital camera (Olympus).

### Carbohydrate quantification

Carbohydrate contents in the purified attractants were quantified using the phenol-sulfuric acid method as described in Nielsen (2017). Approximately 500 µl of 5% w/w phenol were mixed with 500 µl of 0.1, 1, and 10 mg/ml of attractant solutions and glucose standard solutions (10, 20, 50, 80, and 100 µg/ml). Approximately 500 µl of phenol and 2.5 ml of concentrated sulfuric acid were then added to each reaction, and the mixtures were incubated at room temperature for at least 20 min. The absorbance at 490 nm for each reaction was measured. The amount of carbohydrates in the purified attractants was extrapolated from the glucose standards.

### Immuno-blotting

Approximately 20, 4, 0.8, and 0.16 µg of freeze-dried SR culture supernatant and purified attractant were blotted on nitrocellulose membranes, along with purified AGP (Tsumuraya et al., 1988; Tsumuraya et al., 2019), polygalacturonic acid (Megazyme P-PGACT), and rhamnogalacturonan-I (Megazyme P-RHAM1) as positive controls for the antibodies. Membrane was incubated in blocking buffer [50 mM Tris pH 7.4, 15 mM NaCl, 0.1% (v/v) Tween-20, 1% (w/v) skim milk] in RT for 1 h with agitation, probed with primary antibodies LM2 (1:1,000, Yates et al., 1996), LM19 (1:500, Verhertbruggen et al., 2009) or CCRC-M36 (1:500, Pattathil et al., 2010) in antibody buffer [blocking buffer with skim milk reduced to 0.1% (w/v)], incubated in RT for 1 h with agitation, then washed three times with TBS-T (blocking buffer without skim milk) for 10 min each in RT. Membranes were then probed with secondary antibodies anti-rat HRP (for LM2 and LM19, 1:5,000, Cytiva) and anti-mouse HRP (for CCRC-M36, 1:5,000, Cytiva) in antibody buffer for 1 h in RT with agitation, then washed three times with TBS-T for 10 min each in RT. Membranes were then incubated with 1 ml of Immobilon Forte Western HRP substrate (Millipore), and chemiluminescence was then imaged.

### Partial acid hydrolysis

Partial acid hydrolysis was performed essentially as described in Shi et al. (2017). Approximately 1 mg of purified attractant dissolved in 900 µl of ddH_2_O was mixed with 100 µl of 5 M HCl or ddH_2_O, then incubated at 80°C for 16 h. Reactions were then neutralized with equimolar NaOH, then dialyzed in ddH_2_O at 4°C overnight using a dialysis membrane with a 14 kDa molecular cut-off (Wako, size 8). Samples were then freeze-dried and re-suspended in ddH_2_O to test for attraction activities.

### Pectin precipitation

Pectin was precipitated from purified attractants using copper(II) acetate treatment as described in Tsumuraya et al. (1988). Approximately 1/6 volume of 7% copper acetate(II) was added to 100 µl SepPak-purified attractant, whereas ddH_2_O was added to mock-treated samples. The reactions were incubated for 1 h at room temperature. The samples were then centrifuged (15,000 rpm, 5 min), and the supernatants were collected. Approximately 3 volumes of ethanol were then added to each reaction, followed by centrifugation (15,000 rpm, 5 min), and the supernatants were discarded. Approximately 1 ml of 80% ethanol was used to re-suspend the pellets. The samples were chilled on ice, then 5 M HCl was added to a final concentration of 0.3 M and agitated for 30 min. After centrifugation (15,000 rpm, 5 min), the supernatants were discarded, and the pellets were re-suspended in 1 ml of 100 mM EDTA. Samples were then dialyzed against 5 mM EDTA for 12 h, followed by ddH_2_O. Samples were freeze-dried and re-suspended in ddH_2_O to test for attraction activity.

### Yariv binding assay

Yariv binding assay was performed essentially as described in Yariv et al. (1962). Yariv reaction solution (60 µl of 5 M NaCl, 20 µl of 2% NaN₃, 80 µl of 1 mg/ml Yariv reagent (β-Glc) (Fujifilm Wako 536-38581), 2 ml of 1% agarose gel) were spread over a glass slide. The agarose was allowed to set, then wells were made in the agarose gel using an aspirator. Approximately 2 µl of SepPak-purified attractant and 2.5 mg/ml gum arabic as a positive control was loaded into the wells. Slide glass was incubated at room temperature with high humidity overnight.

### Enzyme digestion

Approximately 1 mg of freeze-dried SepPak-purified attractant was mixed with reaction solution (1.5 U enzyme, 200 µl of acetate buffer [500 mM pH 4.1, except polygalacturonase 5.5]), adjusted to 1 ml with ddH_2_O then incubated at 40°C for 4 h. Enzymes used include endo-1, 4-beta-D-galactanase (Megazyme E-EGALN), endo-1, 5-alpha-L-arabinanase (Megazyme E-EARAB), and endo-polygalacturonase (Megazyme E-PGALUSP). Reactions were then terminated by incubation at 100°C for 5 min. Samples were then centrifuged (15,000 rpm, 5 min), and the supernatants were collected and dialyzed twice against sterile water for 12 h each. Samples were freeze-dried and re-suspended in ddH_2_O to test for attraction activity.

## Results

### SR secretes multiple RKN attractants into the environment

**Figure 1:**
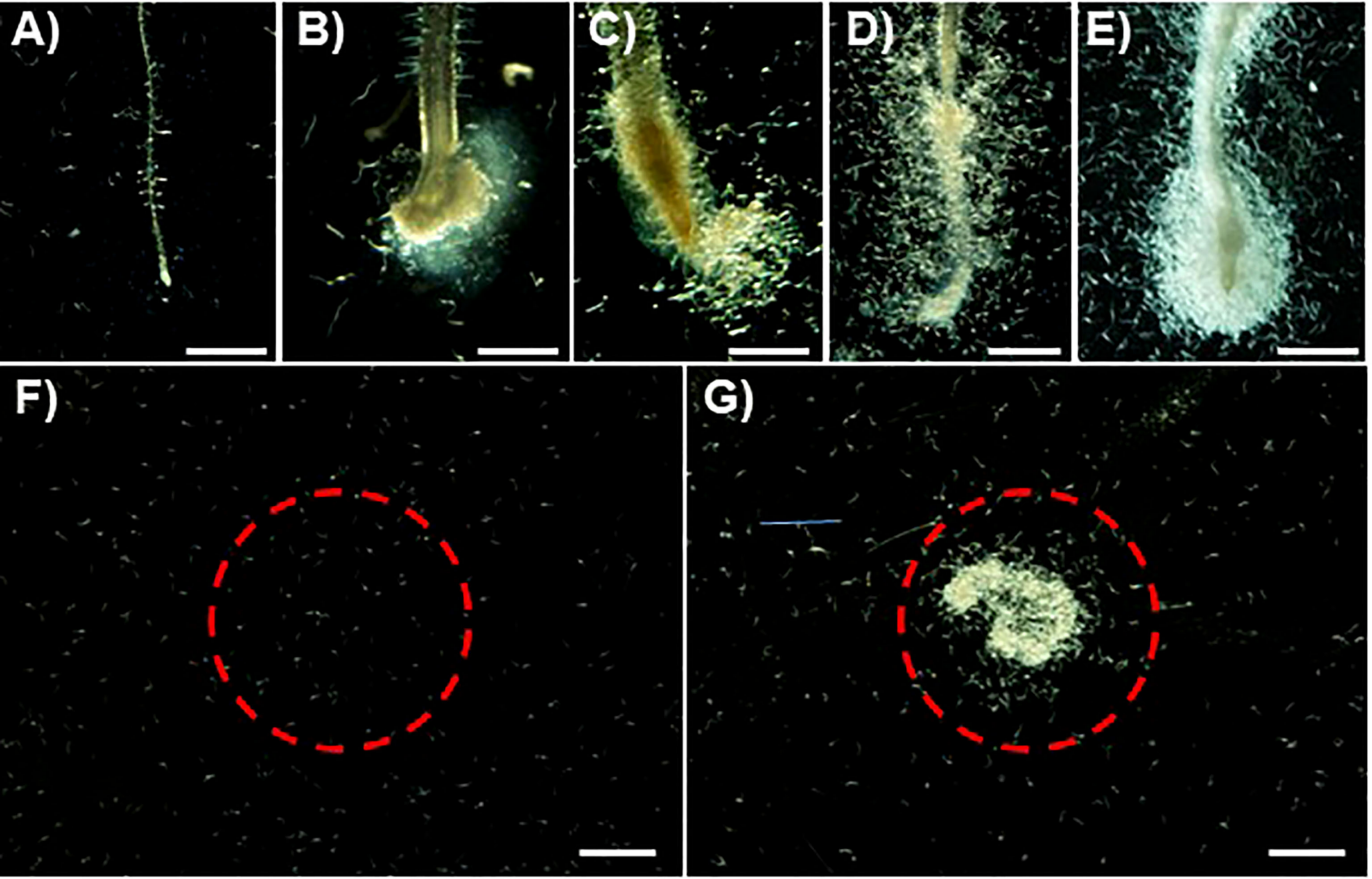
SR secretes RKN attractants to the environment. **(A–D)** Representative RKN behavior *in vitro* in the presence of Arabidopsis **(A)**, rice **(B)**, tomato **(C)**, *L. coniculatus* L. **(D)** roots, and SR **(E)**. **(F, G)** Representative images of RKN behavior *in vitro* in the presence of SR culture media supernatant before **(F)** and after **(G)** SR growth. Red circles denote the area where samples were applied. Scale bars = 1 mm.

To identify a suitable plant species model to characterize RKN chemotaxis, RKN behavior near plant root tips was examined for Arabidopsis (ecotype Col-0), rice (*O. sativa*, cultivar Taichu65), tomato (cultivar Pritz), the legume model plant *L. corniculatus* L., and the super-growing root (SR) culture consisting of excised *L. corniculatus* L. roots (**Figures 1A–E**). Very few RKN J2 larvae were attracted to Arabidopsis root tips, while rice, tomato, and *L. corniculatus* L. roots attracted modest amounts of RKN J2 larvae (**Figures 1A–D**). Surprisingly, SR culture roots attracted substantially more RKN J2 larvae than others (**Figure 1E**). Based on these findings, the RKN-attracting properties of SR culture were further evaluated. To determine whether SR secretes RKN attractants into the environment, the RKN attraction activities of the culture media supernatant used to propagate SR were examined. The culture media used to propagate SR contain markedly higher RKN attraction activity compared to liquid media before SR culturing (**Figures 1F, G**), confirming that SR is indeed likely to secrete RKN-attracting substances into the environment.

**Figure 2:**
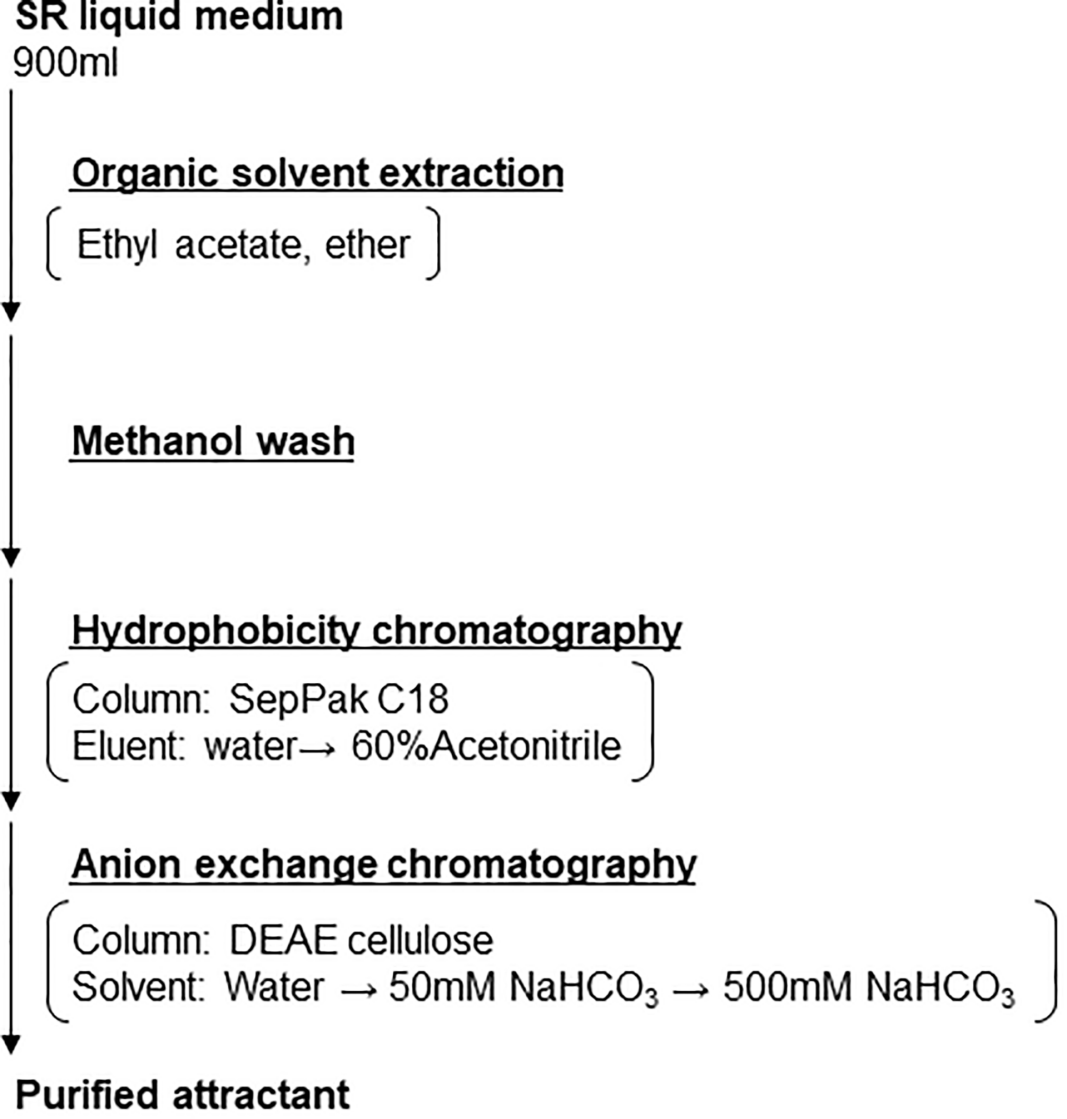
SR-secreted RKN attractant purification strategy. Schematic depicting the purification strategy to enrich and purify the RKN-attracting compounds from SR media supernatant.

**Figure 3:**
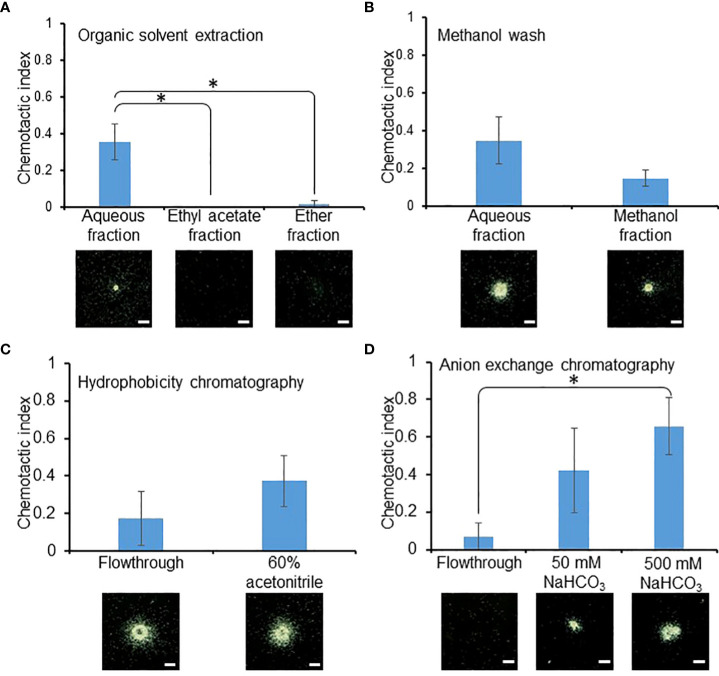
RKN attraction activities were maintained through enrichment. RKN chemotactic indexes of the intermediate fractions during attractant purification from SR media supernatant, during organic solvent extraction **(A)**, methanol wash **(B)**, hydrophobicity chromatography **(C)**, and anion exchange chromatography **(D)**. Averages from n = 3 ± SD are shown. * denotes significant difference between the indicated samples. (student’s T-test, P<0.05). Bottom panels show representative RKN *in vitro* behavior; scale bars = 1 mm.

To identify the RKN attractant secreted by SR, a series of fractionation and precipitation steps were implemented to purify the RKN attractant from the SR culture media supernatant (**Figure 2**). Ethyl acetate and ether were first used to broadly isolate apolar organic compounds from the culture media supernatant. The attraction activity was found to be significantly stronger in the aqueous fraction compared to the ethyl and ether fractions, suggesting the attractant most likely consists of water-soluble molecules (**Figure 3A**). The aqueous fraction was then washed with methanol to further separate the compounds based on their solubility. Surprisingly, both the methanol wash and aqueous fraction contained RKN-attracting activities, suggesting there may be at least two classes of RKN attractants in the SR culture media supernatant with different polarities (**Figure 3B**). Since the attraction activity of the aqueous fraction was stronger (although not significant) than that of the methanol fraction, the aqueous fraction was further investigated in this study. The attractant was then further purified through hydrophobicity and anion exchange chromatography, from which the attractant could be eluted with 60% acetonitrile and bicarbonate, respectively (**Figures 3C, D**). The final purified attractant was eluted with 50 mM and 500 mM NaHCO_3_. Since the 500 mM NaHCO_3_ elution showed significantly stronger attraction activity than the flow-through, only this fraction was chosen for further analysis.

### SR-secreted RKN attractant contains pectin

Root exudates are known to contain significant amounts of carbohydrates (Knee et al., 2001; Cannesan et al., 2012), therefore the SR-secreted RKN attractant is believed to likely be a carbohydrate derivative as well. To test this hypothesis, the phenol-sulfuric acid method was used to quantify carbohydrate levels in the purified SR-secreted RKN attractant (Nielsen, 2017). Using the standard curve in **Figure S1**, 6.08 mg of carbohydrates were detected from 10 mg of purified attractant. This suggests carbohydrates make up >60% (w/w) of the total purified SR RKN attractant, and it is likely that the RKN attractant molecule may be a carbohydrate, possibly a cell wall polymer.

**Figure 4:**
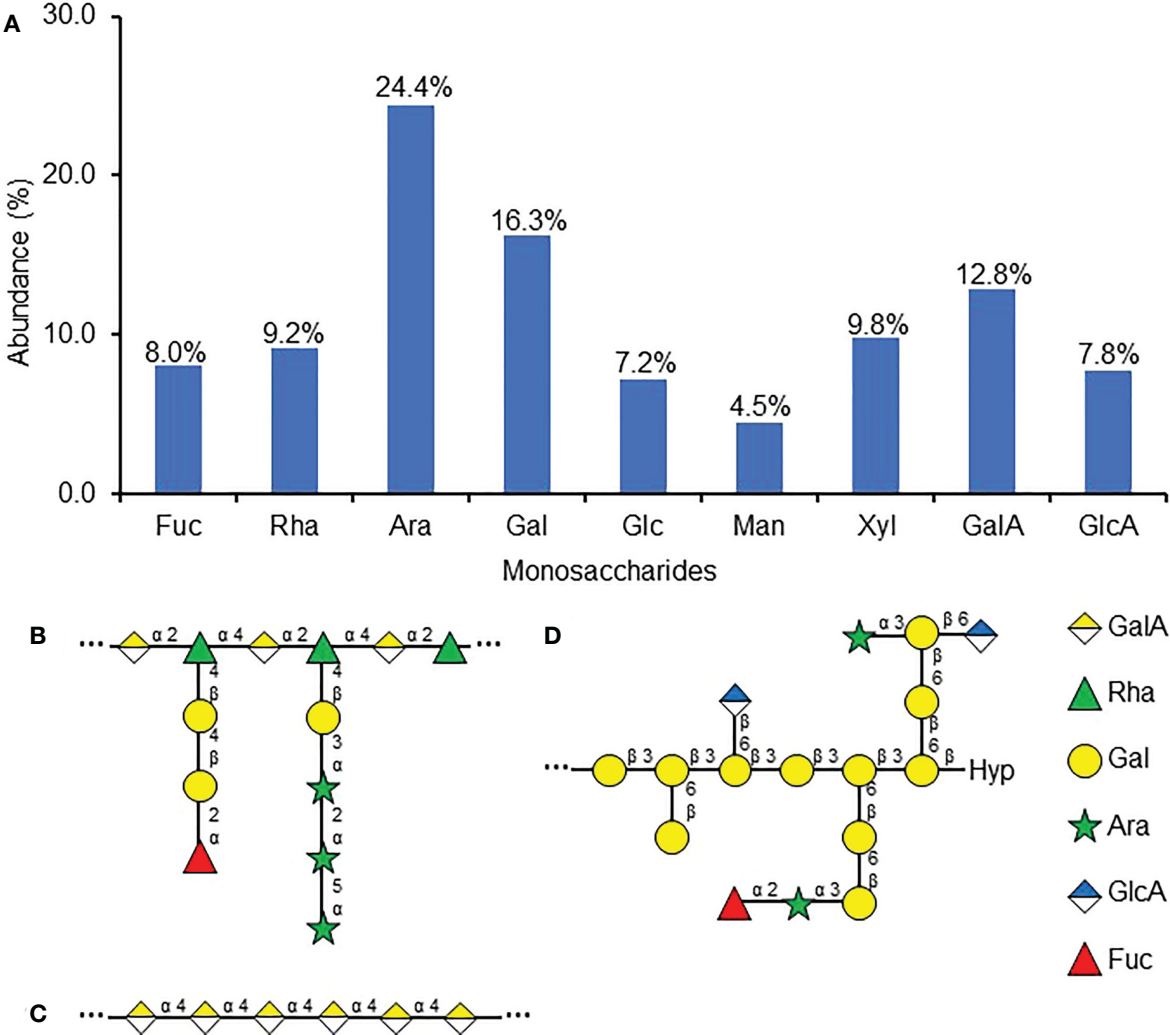
Monosaccharide composition of purified RKN attractant from SR culture media. **(A)** Relative abundances of monosaccharides detected in the purified RKN attractant from SR culture media. Numbers denote the numerical values of fucose (Fuc), rhamnose (Rha), arabinose (Ara), galactose (Gal), glucose (Glc), mannose (Man), xylose (Xyl), galacturonic acid (GalA), and glucuronic acid (GlcA). **(B–D)** Representative structures of cell wall polysaccharides that may be present in the purified attractant based on the monosaccharide analysis, including RG-I (adapted from Mohnen, 2008) **(B)**, HG (adapted from Mohnen, 2008) **(C)**, and AGP (adapted from Tryfona et al., 2012) **(D)**, Hyp, hydroxyproline. “…” denotes that the backbone structures repeat. The right panel shows the symbols used to represent the monosaccharides.

To further characterize the carbohydrates involved in RKN attraction, the monosaccharide composition of the purified attractant was examined. The most abundant monosaccharides present in the purified attractant include arabinose (Ara, 24.4%), galactose (Gal, 16.3%), and galacturonic acid (GalA, 12.8%) (**Figure 4A**). Most other monosaccharides tested, including fucose (Fuc), rhamnose (Rha), glucose (Glc), xylose (Xyl), and glucuronic acid (GlcA), each make up about ~10% of the purified attractant carbohydrate, while mannose (Man) is the least abundant at 4.5% (**Figure 4A**). The prominence of Ara, Gal, and GalA seems to suggest the predominant polysaccharides within the purified attractant may be pectin derivatives, as GalA forms the backbones of rhamnogalacturonan-I (RG-I, carbohydrates with repeating -GalA-α(1,2)-Rha-α(1,4)-backbones, **Figure 4B**) and homogalacturonan (HG, carbohydrates with repeating α(1,4)-linked galacturonan backbones, **Figure 4C**), while Ara and Gal are commonly found in class II arabinogalactans (AG, β(1,3)-linked galactan polymers decorated with arabinan sidechains, **Figure 4D**) and arabinogalactan proteins (AGP, peptides covalently-linked with AG, **Figure 4D**) (Mohnen, 2008; Villa-Rivera et al., 2021). However, the presence of a diverse range of other monosaccharides implies the purified attractant may still contain multiple classes of cell wall polysaccharides.

**Figure 5:**
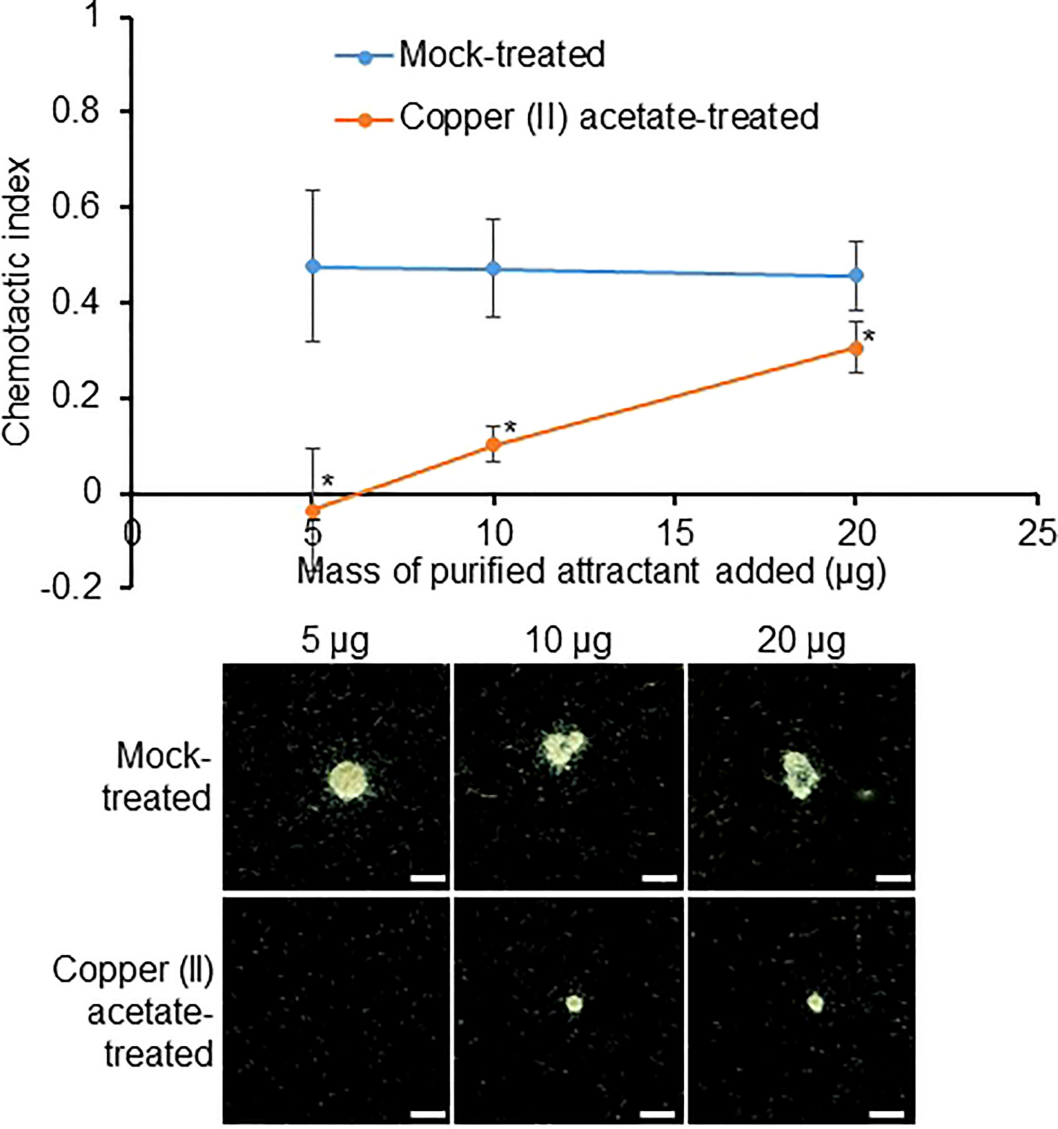
The active compound in the RKN attractant purified from SR culture media may be pectin. RKN chemotactic indexes of attractant purified from SR culture media treated with copper(II) acetate to precipitate pectin. Averages from n = 3 ± SD are shown. *denotes significant differences from mock-treated samples, P<0.05, student’s T-test. Bottom panels show representative RKN behavior *in vitro* in the presence of the attractant, scale bars = 2 mm.

To confirm whether pectins make up the active molecule in the purified RKN attractant, copper(II) acetate was used to precipitate pectin (carbohydrates with charged backbones, essentially RG-I and HG) from the purified RKN attractant (Tsumuraya et al., 1988). Interestingly, copper(II) acetate-treated purified RKN attractant showed significantly reduced RKN attraction activities compared to mock-treated samples (**Figure 5**). With increasing amounts of attractant added, the copper(II) acetate binding capacity gradually became saturated, and attraction strength increased even in the presence of copper(II) acetate (**Figure 5**). This confirms that the molecules responsible for RKN attraction are indeed pectin derivatives.

**Figure 6:**
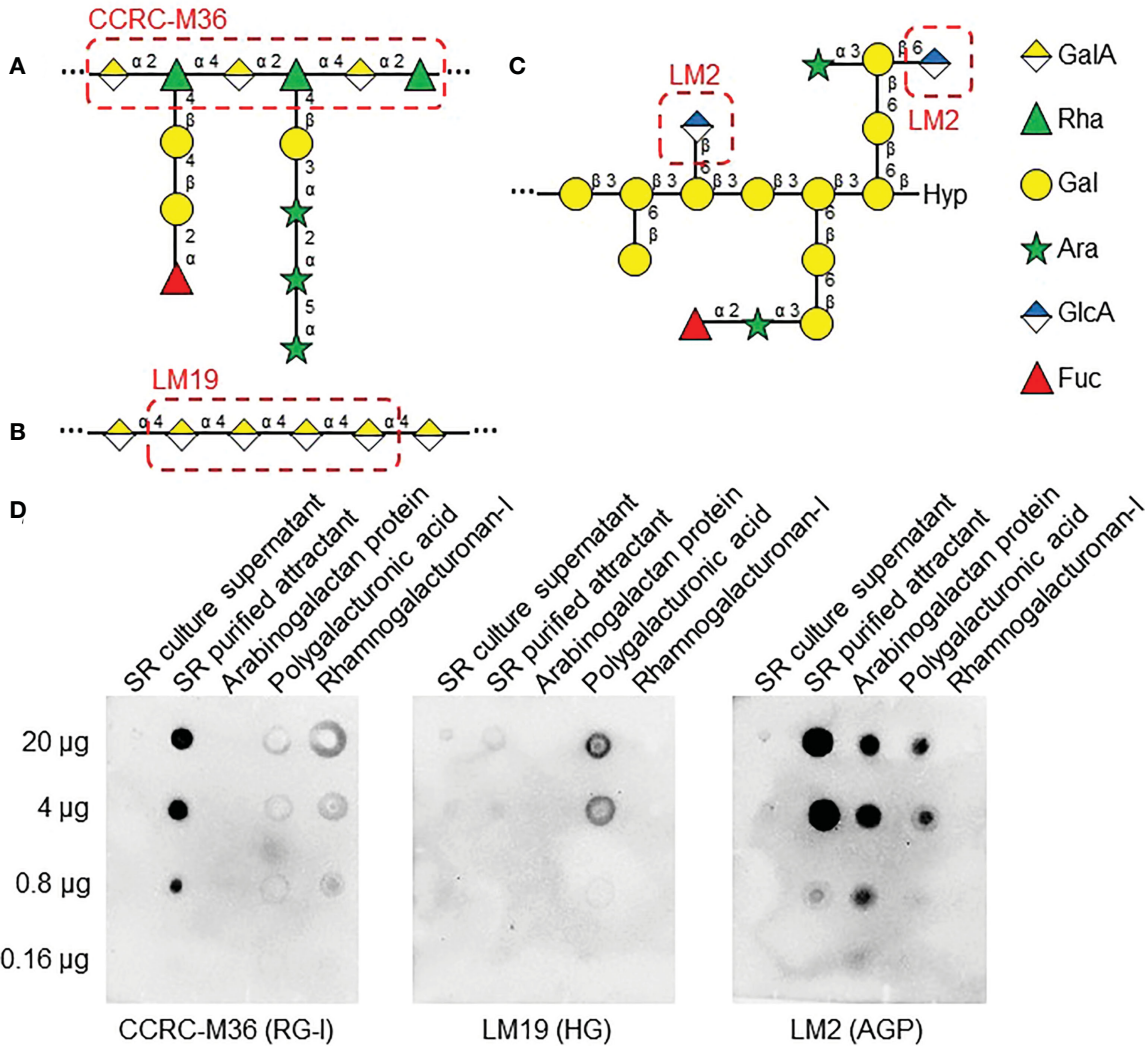
AGP and RG-I epitopes are present in RKN attractants from SR exudates. **(A–C)** Cell wall polysaccharide structures from **Figure 4**, with epitopes of antibodies used in **(D)** highlighted in red dashed boxes. RG-I -GalA-α(1,2)-Rha-α(1,4)- backbone is recognized by CCRC-M36 **(A)**, HG α(1,4)-linked GalA backbone is recognized by LM19 **(B)**, and β-linked GlcA of class II AG is recognized by LM2 **(C)**. **(D)** Immuno-blotting analysis of SR culture supernatant and SR purified RKN attractant, along with purified radish AGP, polygalacturonic acid, and RG-I as positive controls. 5-fold serial dilutions, starting with 20 µg of carbohydrates, were used as substrates. Samples were probed with LM2 (anti-AGP, left panel), LM19 (anti-HG, middle panel), and CCRC-M36 (anti-RG-I, right panel) antibodies.

Next, to determine which class of pectin is present in SR RKN attractant, freeze-dried SR culture supernatant and purified attractant were probed with cell wall carbohydrate antibodies CCRC-M36 [immunized with RG-I, recognizes Rha-(1,4)-GalA-(1,2)-] (**Figure 6A**), LM19 (immunized with HG, recognizes 1, 4-linked α-GalA) (**Figures 6B**), and LM2 (immunized with AGP, recognizes β-linked GlcA) (**Figure 6C**) through immuno-blotting analysis (Yates et al., 1996; Verhertbruggen et al., 2009; Pattathil et al., 2010). LM2 and CCRC-M36 epitopes were strongly enriched in the purified attractant, confirming it contains RG-I backbones and class II AG (**Figure 6D**). On the other hand, LM19 is barely detectable in the purified attractant, suggesting the attractant is unlikely to contain HG (**Figure 6D**). All three antibodies were able to detect their respective cognate cell wall carbohydrates.

### Substituted RG-I is responsible for RKN attraction in SR exudates

Considering the anti-AGP LM2 epitopes were detected in the purified attractant, the possibility of the attractant being AGPs was investigated first. AGPs are heavily glycosylated members of the hydroxyproline-rich glycoprotein superfamily and are known to be expressed in various root tissues and secreted in root exudates (Cannesan et al., 2012; Nguema-Ona et al., 2013, **Figure 4D**). Interestingly, AGPs in root exudates have been shown to attract the zoospores of the root pathogenic oomycete *Aphanomyces euteiches* (Cannesan et al., 2012; Laloum et al., 2021). To test whether the purified RKN attractant contained AGPs, it was probed with β-Yariv reagents, which are known to selectively bind the β-(1,3)-linked galactan backbones of AGPs (Yariv et al., 1962; Kitazawa et al., 2013; **Figure S2A**). When samples that contain AGP are blotted on β-Yariv-imbued agarose gel, AGP is stained in a deep red color where the samples were applied (**Figure S2B**, top panels). However, similar stains were not observed when a purified attractant was applied, suggesting the purified attractant is unlikely to contain β-(1,3)-linked galactan or AGPs (**Figure S2B**, bottom panels). To further evaluate the relationships between AGPs and RKN attraction, two types of AGPs extracted from radish (*Raphanus raphanistrum* subsp. *sativus*) and pear (*Pyrus* spp.) (Tsumuraya et al., 1988; Tsumuraya et al., 2019) were tested for RKN-attracting activities (**Figure S2C**). Neither of the AGPs tested produced visible RKN colonies at up to 20 mg/ml and showed generally very low attraction indices (**Figure S2C**). However, AGPs are exceptionally diverse in structure and biological functions (Seifert and Roberts, 2007). Since anti-AGP LM2 epitopes are present in the purified attractant, it is still possible for other AGPs to play a role in RKN chemotaxis.

The anti-RG-I CCRC-M36 epitopes were also present in the purified attractant; the molecules responsible for RKN attraction in SR exudates may also be RG-I. GalA and Rha, which make up the backbones for most of the pectin classes, are present in the purified RKN attractant (**Figure 4A**). Since the GalA and Rha levels are roughly comparable, RG-I with the backbone monosaccharide Rha : GalA ratio of roughly 1:1 is likely the main pectin species in the purified attractant (**Figure 4B**). RG-I has also been found to be covalently linked with arabinan and galactan sidechains (Mohnen, 2008, **Figure 4B**), justifying the detection of high levels of Ara and Gal in the purified RKN attractant. Interestingly, pectin has been associated with nematode chemotaxis before. Previously, flax seed coat mucilage RG-I was identified as an RKN attractant, where the L-Gal sidechains are essential for attraction (Tsai et al., 2021).

**Figure 7:**
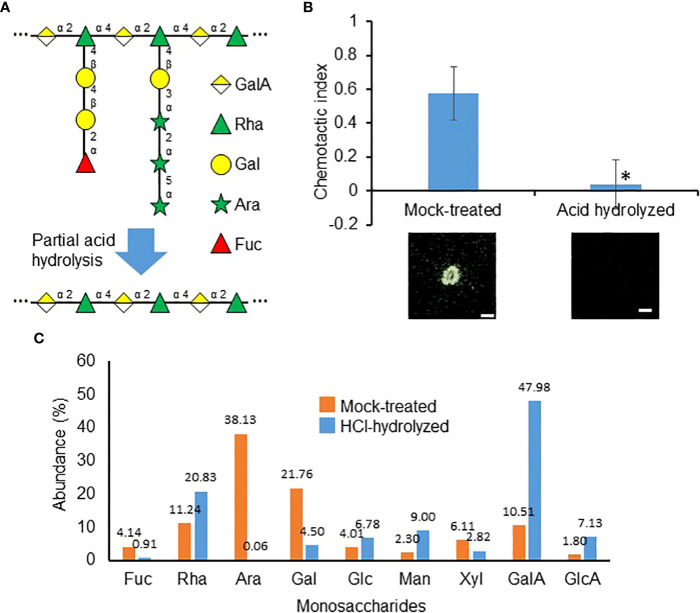
Carbohydrate sidechains are important for the purified attractant. **(A)** Representative RG-I structure from **Figure 4B** to depicting the theoretical loss of sidechains during partial acid hydrolysis. **(B)** RKN chemotactic indexes of attractant purified from SR culture media partially hydrolyzed with HCl. Averages from n = 3 ± SD are shown. *denotes significant differences from mock-treated samples, P<0.05, student’s T-test. Bottom panels show representative RKN behavior in the presence of the attractant, scale bars = 1 mm. **(C)**. Relative abundances of monosaccharides detected in mock-treated and HCl-hydrolyzed purified attractants. Numbers denote the numerical values of fucose (Fuc), rhamnose (Rha), arabinose (Ara), galactose (Gal), glucose (Glc), mannose (Man), xylose (Xyl), galacturonic acid (GalA), and glucuronic acid (GlcA).

To determine whether RG-I sidechains similarly play a role in the purified RKN attractant, partial hydrolysis was performed with hydrochloric acid to preferentially remove these sidechains (**Figure 7A**). Intriguingly, partial hydrolysis significantly reduced the RKN attraction activities in purified attractants, suggesting the Ara and Gal sidechain groups are indeed required for attraction activities (**Figure 7B**). Partial hydrolysis indeed reduced the Ara and Gal levels in the purified RKN attractants, while Rha and GalA levels increased, confirming the sidechains have been preferentially removed (**Figure 7C**). To more precisely determine the sidechain functional groups associated with RKN attraction from SR, polygalaturonase (Megazyme E-PGALUSP, catalyzes random hydrolysis α-1,4-D-galactosiduronic linkages in HG and RG-I), arabinanase (Megazyme E-EARAB, catalyzes endo-hydrolysis of (1,5)-α-arabinofuranose linkages in (1,5)-α-arabinans) and galactanase (Megazyme E-EGALN, catalyzes endo-hydrolysis of (1,4)-β-D-galactose linkages in (1,4)-β-galactans and type I arabinogalactans) were used to treat the purified attractant in an attempt to abolish its attraction activities (**Figure S3**). However, none of these enzymes, individually or in permutations of their mixtures, significantly affected the RKN-attracting activities of the purified attractant (**Figure S3**). The sidechain groups and linkages involved in RKN attraction by SR remain to be elucidated.

Lastly, we were also interested in testing whether the RKN attractant purified from SR culture supernatant also attracted other nematodes. However, neither *M. enterolobii*, *M. arenaria*, nor *C. elegans* showed chemotactic behavior toward SR culture media supernatant, unlike *M. incognita* (**Figure S4**). This suggests *M. incognita* may have capitalized on the perception of a wide array of cell wall polysaccharides as a host-targeting strategy.

## Discussion

### Pectin-derivatives secreted by SR attracts RKN

Here we describe the isolation and characterization of RKN-attracting compounds secreted by *L. corniculatus* L. SR cultures, and one of these RKN attractants is composed of derivatives of pectinacious cell wall carbohydrates. Cell wall carbohydrate-based signaling molecules have been documented in the past, with the wall-associated kinase (WAK) pathway being the best studied (Kohorn, 2016). Both cross-linked pectins and short homogalacturonan fragments have been shown to bind WAK homologues, possibly induced cell expansion and pathogen responses, respectively (He et al., 1996; Lally et al., 2001; Decreux and Messiaen, 2005; Denoux et al., 2008). Similarly, cell wall carbohydrate-based RKN-attracting molecules have also been identified before. Arabidopsis seeds were found to attract RKN in a mucilage extrusion-dependent manner (Tsai et al., 2019), and the L-Gal-Rha linkage found in flax seed coat mucilage RG-I was also found to be essential for attracting RKN (Tsai et al., 2021). It is thus perhaps not surprising to find other pectin-based RKN attractants secreted by plants. Nevertheless, this marks the first instance of a carbohydrate-based attractant being secreted from the roots. Since the levels of Rha and GalA appear relatively comparable, the predominant pectin in the attractant is most likely to be RG-I, where the Rha and GalA ratio is predicted to be 1:1 (**Figure 4B**). However, since GalA may be underestimated due to incomplete hydrolysis, in reality, GalA levels may be higher (De and Timell, 1967; Shi et al., 2020). The presence of excess GalA implies the presence of HG is possible, though the lack of LM19 epitopes in the purified attractant suggests it is unlikely (**Figures 4C**, **6D**).

The purified RKN attractant is sensitive to partial acid hydrolysis, similar to flax RG-I, implying that the polysaccharide sidechains are essential for attraction (Tsai et al., 2021). Unlike flax RG-I, the sidechains cleaved off by partial acid hydrolysis in SR-secreted RKN attractant contain primarily Gal and Ara. In flax mucilage RG-I, the sidechains consist of single Gal or Fuc residues (Naran et al., 2008; Tsai et al., 2021). In contrast, the sum of Ara and Gal exceeds the sum of GalA and Rha in the purified RKN attractant from SR. Therefore, sidechains in SR are more likely to be complex polymeric AGs instead of single residues.

AG refers to polysaccharides with galactan backbones decorated with arabinan sidechains. Currently, three classes of AG have been categorized based on their galactan backbone linkages: β-(1,4), β-(1,3) and β-(1,6), with AGPs being associated with β-(1,3)-linked galactans (Villa-Rivera et al., 2021) (**Figure 4D**). AGPs have not only been shown to attract oomycetes zoospores (Cannesan et al., 2012; Laloum et al., 2021), but also aid the attachment to endophytic *Rhizobium* and *Agrobacterium* on plant roots (Gaspar et al., 2004; Vicré et al., 2005). Many rhizosphere microbes also secrete enzymes specifically to digest AGP carbohydrates to consume as a nutrient source (Knee et al., 2001). Suffice to say AGPs play critical roles in regulating interactions between both symbiotic and pathogenic microorganisms in the rhizosphere. The fact that the purified RKN attractant contains LM2 epitopes but did not bind to the β-Yariv reagent is thus somewhat surprising, even though the two epitopes do not necessarily both occur in all AGPs (**Figure 6D**, **Figure S2B**). Other root exudates that contain AGPs, as well as AGPs precipitated from root exudates using β-Yariv share similar monosaccharide profiles with the purified RKN attractant (**Figure 4A**; Knee et al., 2001; Cannesan et al., 2012). In addition, Tan et al. (2013) have suggested that the AGP carbohydrate moieties are the same molecules as the AG sidechains of RG-I, such that AGP and RG-I are in fact covalently linked together as a single macromolecule. These lines of evidence make it difficult to confidently reject AGP as the compound responsible for RKN attraction in SR. It is possible that the AGs in the purified attractant possess either β-(1,4) or β-(1,6)-linked galactans, however unlike β-(1,3)-linked galactans these other AG classes have yet to be shown to regulate plant–pathogen interactions (Nguema-Ona et al., 2013; Villa-Rivera et al., 2021). Currently, the only line of evidence against the presence of AGPs in the purified RKN attractant in SR is the lack of β-Yariv reagent binding; linkage analysis of the purified RKN attractant is thus needed to comprehensively determine its structure and the presence of AGPs. The fact that radish and pear AGPs do not attract RKN suggests that if the SR exudate attractant is indeed an AGP, it most likely contains unique structural features essential for attraction.

In addition, RKN attraction activities were also detected in the methanol wash fraction during purification (**Figure 3B**). It is therefore highly likely that RKN perceive and respond to multiple compounds secreted by SR. Multiple RKN repellants have also been isolated from root exudates of various species based on polarities, reinforcing the notion that root exudates are chemically complex (Wang et al., 2018). Some chemicals in root exudates, such as lauric acid from the crown daisy (*Chrysanthemum coronarium* L.), can be a RKN attractant or repellant depending on the concentration (Dong et al., 2014). Lastly, RKN may also respond to chemical cues secreted by other microorganisms in the rhizosphere. Dibenzofuran secreted by *Streptomyces plicatus* G. attracts RKN, yet benzothiazole secreted by the same microbe repels RKN (Wang et al., 2019). These lines of evidence are in line with the hypothesis that RKN chemotactic behaviors are fine-tuned by multiple stimuli to more precisely guide RKN to the site of invasion (Tsai et al., 2020). The identification and characterization of the methanol-soluble attractant will thus help paint a significantly more comprehensive picture of RKN chemotaxis toward SR.

### Potential of SR as a new RKN infection model

Plant root cultures such as the hairy root system and SR have the potential to investigate new aspects of RKN research as well as mass-produce compounds of interest (Gantait and Mukherjee, 2021). Hairy root culture (HRC), i.e., roots transformed by *Agrobacterium rhizogenes*, can indeed be infected by arbuscular mycorrhizal fungi and cyst nematodes, suggesting they can be utilized to investigate plant–microbe interactions (Mugnier and Musse, 1987; Cai et al., 1997). HRC of cucumber (*Cucumis sativus*), tomato (*S. lycopersicum*), and plants in the genus *Prunus* have been used to maintain RKN populations and study their interaction with susceptible and resistant plants (Bosselut et al., 2011; Iberkleid et al., 2015; Díaz-Manzano et al., 2016). However, HRC requires *Agrobacterium*-induced phytohormone production. SR makes it possible to avoid these changes in the hormonal balance and thus more closely resembles wild-type roots under natural conditions. SR was discovered in a line of *L. corniculatus* L. with particularly high root growth activity, capable of producing more root tissues in less time than other lines (Akashi et al., 1998; Akashi et al., 2003). SR appears to be able to subculture essentially indefinitely using only one culture medium, and regenerate plants and protoplasts that retain enhanced root growth activities (Akashi et al., 1998; Akashi et al., 2003). Furthermore, SR can be transformed using *A. rhizogenes* and *A. tumefaciens* and can carry out phenotypic screens (Tanaka et al., 2008; Jian et al., 2009; Himuro et al., 2011). These features make SR a very appealing system for studying various aspects of plant roots.

However even though the plant tissues generated from SR culture superficially resemble WT plants, currently the molecular mechanism behind SR’s enhanced root growth activities remains unclear. Even though the wild-type *L. corniculatus* L. root also attracts RKN (**Figure 1D**), further analyses are required to determine whether the attraction strengths of the SR culture are enhanced compared to those of the wild-type *L. corniculatus* root. It is possible that the production of pectin-based RKN attractants is associated with the SR phenotype and not biologically relevant. Nevertheless, SR may be cultured on industrial scales and mass-produce the RKN attractant to be used directly to control RKN infections in fields. Currently effective and environmentally friendly RKN control strategies remain lacking, making plant-synthesized RKN attractants an appealing approach to protect crop plants in agriculture. Further genetic analysis may help delineate the biosynthesis mechanism of this pectin-based RKN attractant in SR.

Finally, *L. corniculatus* L., a forage crop, is closely related to the model legume *Lotus japonicus*, which has been instrumental in deciphering the molecular genetics of *Rhizobium* root nodulation in legume crops. Both *Lotus* species have been documented to be susceptible to RKN infection (Lohar and Bird, 2003; Moye Jr. et al., 2018). Furthermore, *L. japonicus* genes involved in nodulation have been shown to also affect RKN infection (Lohar and Bird, 2003), suggesting RKN and Rhizobia infection pathways likely share overlapping signaling modules. There may also be merit in examining whether *L. japonicus* roots also secrete the same pectin based RKN attractant, as well as whether RKNs are able to complete their life cycle on *L. corniculatus* L. SR culture. By combining the *L. corniculatus* L. SR culture and the *L. japonicus* genetic resources, the genus *Lotus* has great potential in determining the synthesis of RKN attractant, the plant immune response against RKN infection, as well as the relationship between RKN and Rhizobia infection.

## Data availability statement

The original contributions presented in the study are included in the article/**Supplementary Material**. Further inquiries can be directed to the corresponding author.

## Author contributions

MO, ST, and SS conceived and designed the experiments. MO, ST, TK, NW, and HI performed the experiments. MH, RA, and BF provided resources. MO, ST, A-LT, and SS wrote the manuscript. HI, BF, A-LT, and SS revised the manuscript. All authors read and approved the final manuscript.

## Supplementary material

Supplementary Figure 1 Phenol-sulfuric acid quantification standard curve Standard curve generated using known quantities of glucose standards to quantify the amount of carbohydrates in the purified RKN attractant from SR culture media supernatant.

Supplementary Figure 2 SR-secreted RKN attractant does not react with β-Yariv reagent **(A)** Representative class II AG structure from Fig. 4B with the β-Yariv reagent epitope, β-1,3-galactopentaose highlighted in red dashed box. **(B)** Purified AGP and RKN attractant purified from SR culture media blotted on β-Yariv reagent-imbued agarose, circular deep red staining denote the presence of AGP in samples. **(C)** Attraction indexes of SR meida, and AGP purified from radish and pear. Averages from n = 3 ± SD are shown. * denotes significant difference from SR media (student’s T-test, P<0.05). Bottom panels show representative RKN behavior in the presence of samples, scale bars = 2 mm.

Supplementary Figure 3 Cell wall active enzymes do not affect RKN attraction activity of purified attractant RKN chemotactic indexes of attractant purified from SR culture media digested single **(A)** or multiple enzymes **(B)** using polygalacturonase, arabinose and galanctanase. Averages from n = 3 ± SD are shown. No statistical significance was detected in digested samples compared to mock-treatments (student’s T-test, P<0.05). Bottom panels show representative RKN behavior in the presence of the attractant, scale bars = 1 mm.

Supplementary Figure 4 Attraction activity of the attractant secreted by SR may be limited to *M. incognita* **(A)** Frequencies of attraction toward SR culture media supernatant for various *Meloidogyne* species. n = 6 for *M. incognita*, 5 for *M. enterolobii*, and 1 for *M. arenaria*. **(B)** Chemotactic indexes of *C. elegans* toward SR culture media supernatant and isoamyl alcohol. Averages from n = 3 ± SD are shown.
