# Supplementary figures and images for: Rhamnogalacturonan-I as a nematode chemoattractant from Lotus corniculatus L. super-growing root culture
Source: Front Plant Sci. 2023 Jan 26;13:1008725. doi: 10.3389/fpls.2022.1008725 (PMC9908596; doi:10.3389/fpls.2022.1008725)

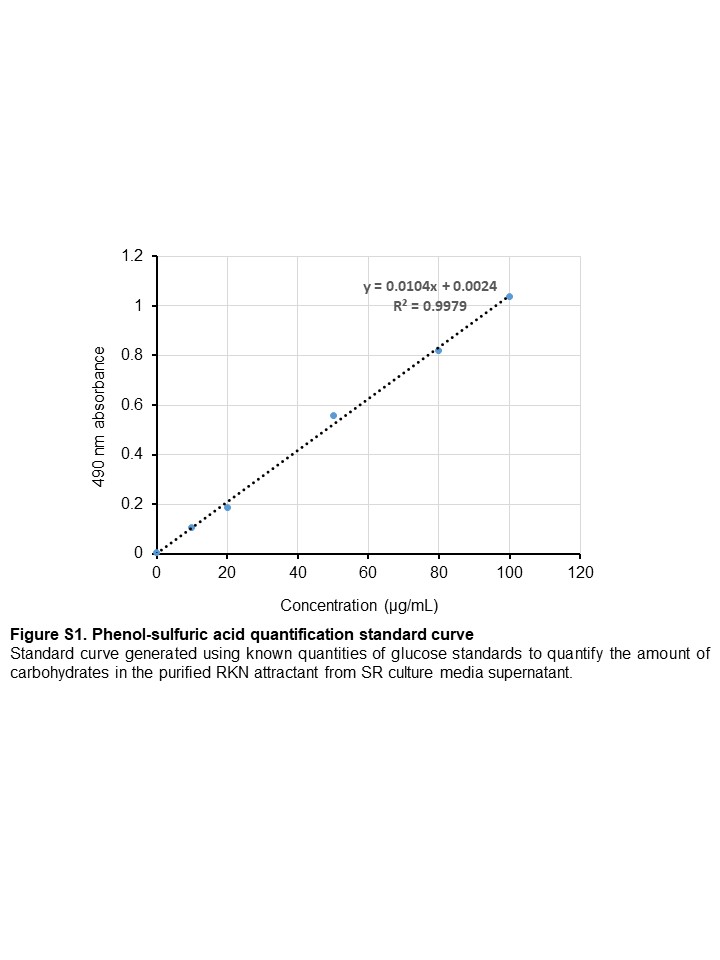

Supplement: Supplementary Figure 1 — Phenol-sulfuric acid quantification standard curve Standard curve generated using known quantities of glucose standards to quantify the amount of carbohydrates in the purified RKN attractant from SR culture media supernatant. [file Image_1.jpeg]

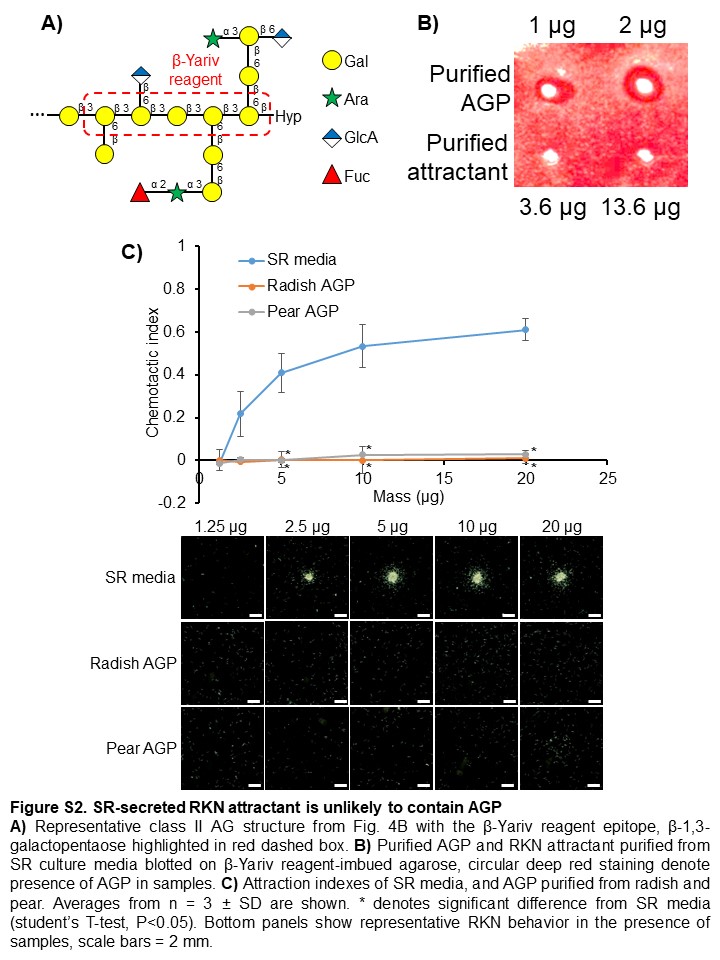

Supplement: Supplementary Figure 2 — SR-secreted RKN attractant does not react with β-Yariv reagent (A) Representative class II AG structure from Fig. 4B with the β-Yariv reagent epitope, β-1,3-galactopentaose highlighted in red dashed box. (B) Purified AGP and RKN attractant purified from SR culture media blotted on β-Yariv reagent-imbued agarose, circular deep red staining denote the presence of AGP in samples. (C) Attraction indexes of SR meida, and AGP purified from radish and pear. Averages from n = 3 ± SD are shown. * denotes significant difference from SR media (student’s T-test, P<0.05). Bottom panels show representative RKN behavior in the presence of samples, scale bars = 2 mm. [file Image_2.jpg]

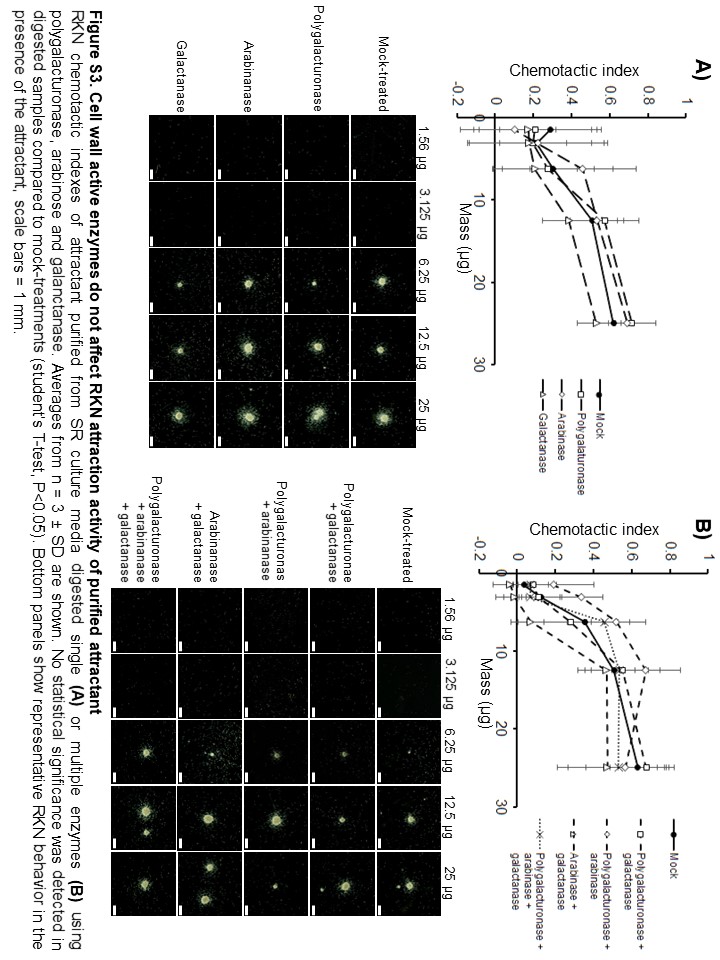

Supplement: Supplementary Figure 3 — Cell wall active enzymes do not affect RKN attraction activity of purified attractant RKN chemotactic indexes of attractant purified from SR culture media digested single (A) or multiple enzymes (B) using polygalacturonase, arabinose and galanctanase. Averages from n = 3 ± SD are shown. No statistical significance was detected in digested samples compared to mock-treatments (student’s T-test, P<0.05). Bottom panels show representative RKN behavior in the presence of the attractant, scale bars = 1 mm. [file Image_3.jpeg]

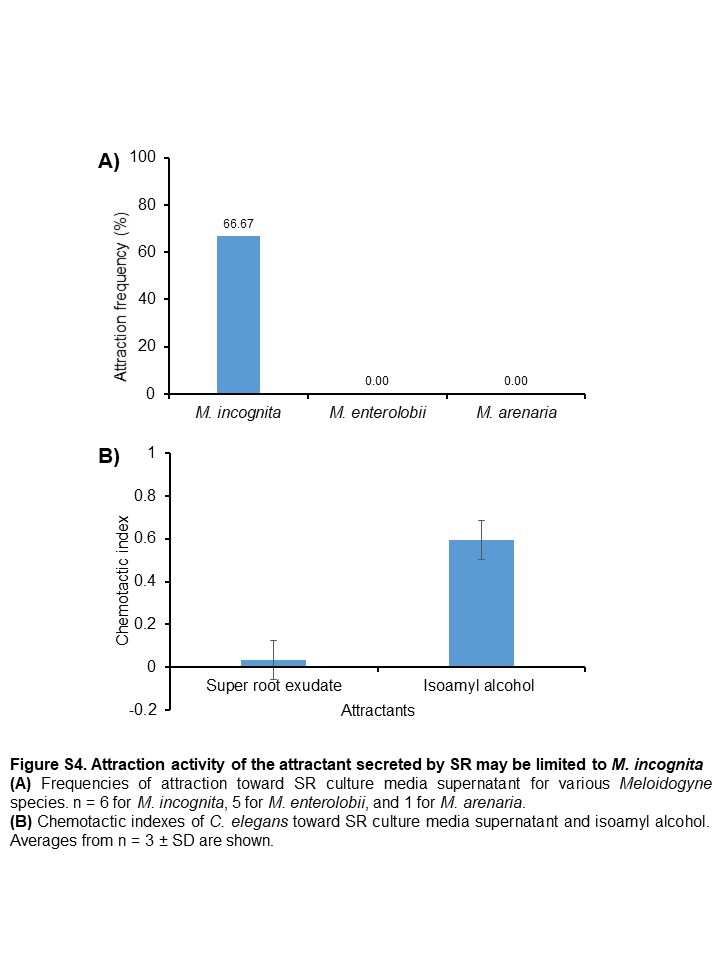

Supplement: Supplementary Figure 4 — Attraction activity of the attractant secreted by SR may be limited to M. incognita (A) Frequencies of attraction toward SR culture media supernatant for various Meloidogyne species. n = 6 for M. incognita, 5 for M. enterolobii, and 1 for M. arenaria. (B) Chemotactic indexes of C. elegans toward SR culture media supernatant and isoamyl alcohol. Averages from n = 3 ± SD are shown. [file Image_4.jpeg]
